# Supplementary material for: Associations with Blood Lead and Urinary Cadmium Concentrations in Relation to Mortality in the US Population: A Causal Survival Analysis with G-Computation
Source: Toxics. 2023 Jan 29;11(2):133. doi: 10.3390/toxics11020133 (PMC9966985; doi:10.3390/toxics11020133)
Supplement: Supplementary file 1 [file toxics-11-00133-s001.zip › toxics-2146836-supplementary.pdf]

toxics

MDPI

# Supplementary Materials: Associations with Blood Lead and Urinary Cadmium Concen-trations in Relation to Mortality in the US Population: A Causal Survival Analysis with G-computation

**Nasser Laouali, Tarik Benmarhnia, Bruce P. Lanphear and Youssef Oulhote**

**Supplementary methods.** The healthy eating index 2015 (HEI-2015) and the adapted dietary inflammatory index (ADII)

The Adapted Dietary Inflammatory Index (ADII) was constructed to measure the inflammatory potential of the diet.[67] Briefly, the ADII index proposed by Woudenberg et al [68] was used in combination with the updated dietary components inflammatory weights designed by Shivappa et al [69] instead of the weights proposed by Cavicchia et al.[70] These updated inflammatory weights, based on 3 additional years of published data (2008–2010, inclusive), resulted in a doubling of the total number of articles scored. The ADII was based on a nutritional rationale: first, the inflammatory weights of dietary components are multiplied by the standardized energy adjusted intake,[71] which acts to reduce between-person variation; second, the intakes of all components are standardized; third, the ADII calculation did not include alcoholic beverages such as beer, wine, and liquor, total fat, and energy, to avoid overestimation of the inflammatory effects of ethanol, fat and energy. A total of 24 of the 35 possible dietary components were used for the ADII calculation (carbohydrates, proteins, alcohol, fibers, cholesterol, saturated fatty acids, monounsaturated fatty acids, omega 3, omega 6, niacin, thiamin, riboflavin, vitamin B6, vitamin B12, iron, magnesium, zinc, vitamin A, vitamin C, vitamin D, vitamin E, folic acid, beta carotene, and caffeine). A positive ADII score indicates an anti-inflammatory diet and negative values correspond to a pro-inflammatory diet.

The Healthy Eating Index 2015 (HEI-2015) was developed to measure overall diet quality.[72,73] Briefly, HEI-2015 is a composite measure of conformance to the 2015–2020 Dietary Guidelines for Americans and has been well validated in the US population.[74] The adequacy components (maximum score) were total fruits (5), whole fruits (5), total vegetables (5), greens and beans (5), whole grains (10), dairy (10), total protein foods (5), seafood and plant proteins (5), and fatty acids (ratio of the sum of polyunsaturated and monounsaturated fatty acids to saturated fatty acids, 10). The moderation components were refined grains (10), sodium (10), added sugars (10), and saturated fats (10). HEI-2015 is a 100-point scale, with a higher score indicating a better quality of overall diet.

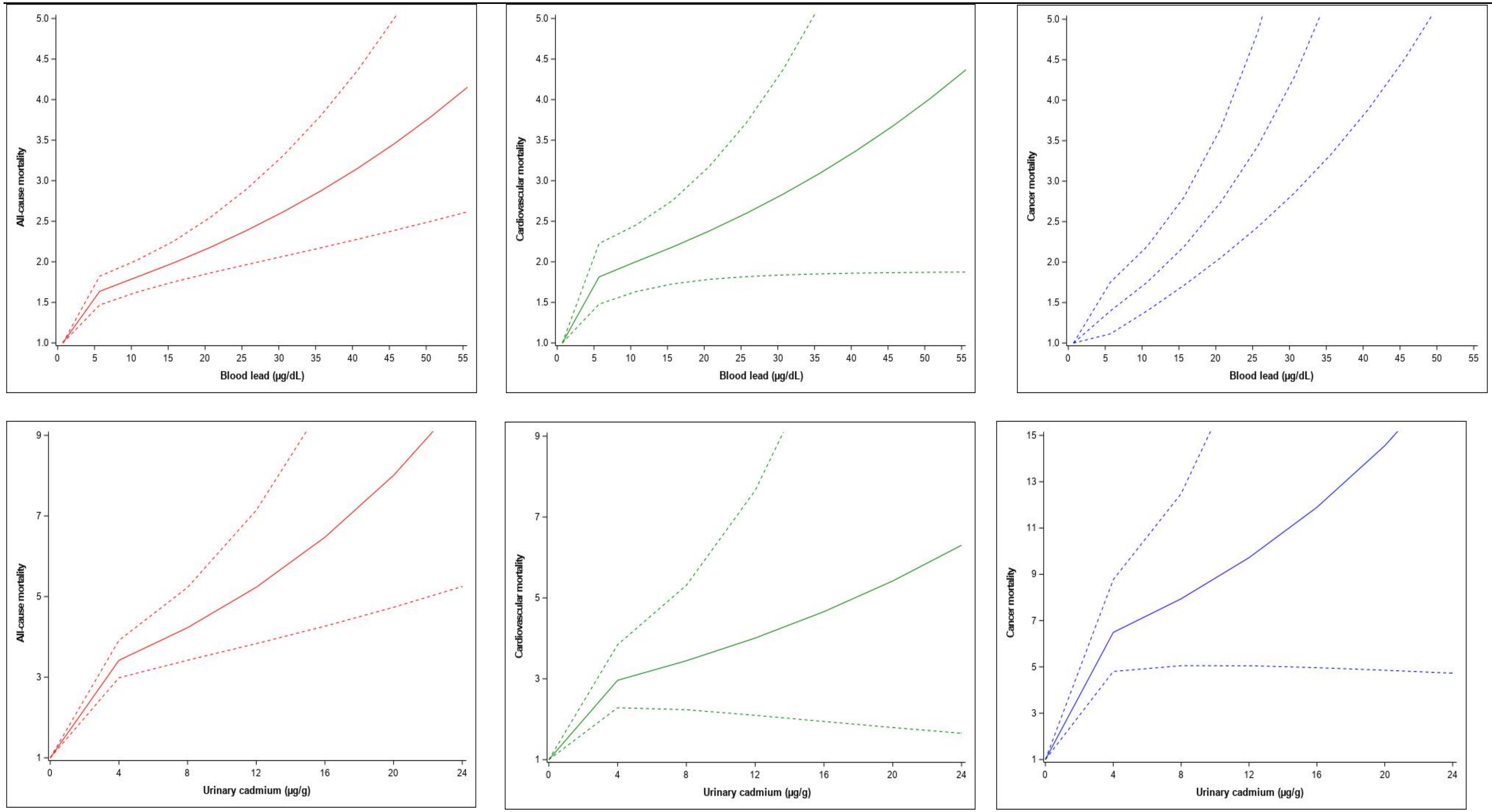


**Figure S1.** Adjusted all-cause (red), cardiovascular disease (green) and cancer (blue) mortality associated to blood lead and urinary cadmium concentrations using restricted cubic spline (three knots placed at the 5th, 50th, and 95th percentiles). Models were adjusted for age, sex, ethnicity, poverty index, educational level, area of residence, smoking status, BMI, physical activity, diet quality evaluated by the healthy eating index and metals concentrations (mutual adjustment). Reference values for HRs: minimum concentrations; the solid lines represent the HR and the dashed lines the lower and upper 95% CI.

**A** Blood lead: Age < 50

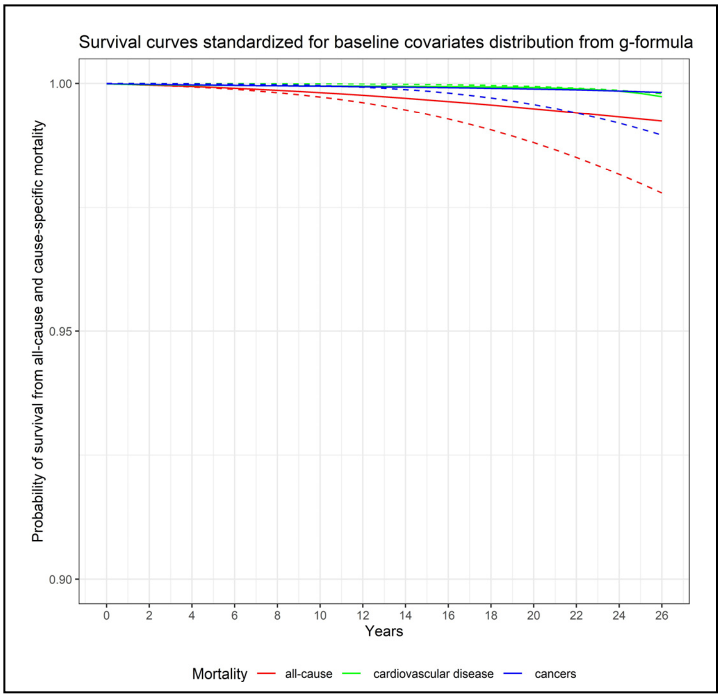


**B** Blood lead: Age ≥ 50

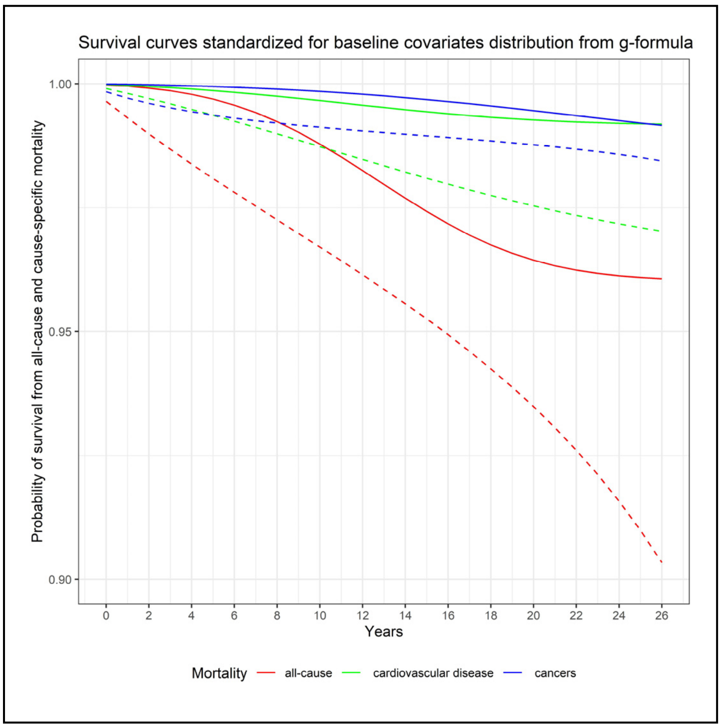


**C** Urinary cadmium: Age < 50

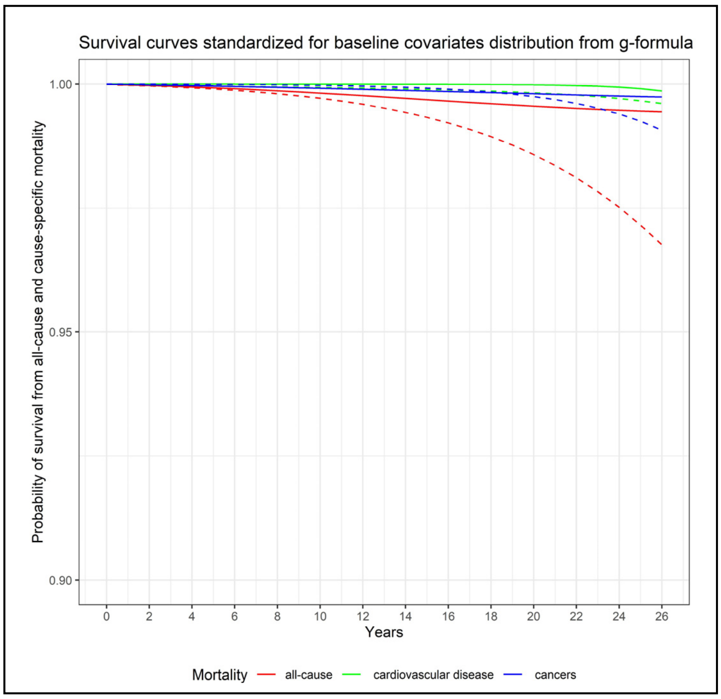


**D** Urinary cadmium: Age ≥ 50

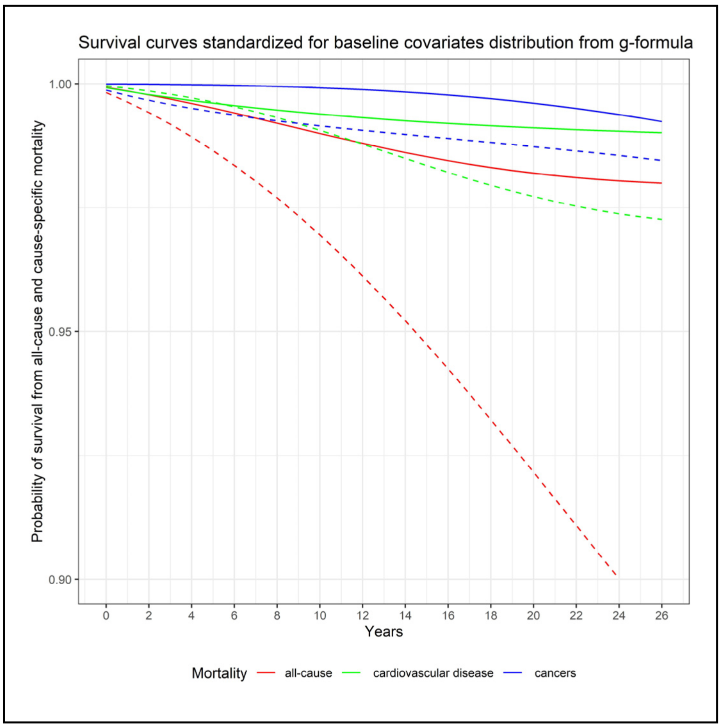


**Figure S2.** Adjusted all-cause and specific-cause of mortality risk stratified by age using parametric g-formula with pooled logistic regression models. The solid line represents the percentile 5 (0.70 µg/dL for lead and 0.04 µg/g for cadmium) and the dashed lines the percentile 95 (9.70 µg/dL for lead and 1.63 µg/g for cadmium). Robust 95% confidence intervals (CIs) for each exposure category estimated by bootstrapping (in the pooled logistic regression model**)** are presented in Table S1.

**Table S1.** Adjusted all-cause and specific-cause of mortality risk ratio and risk difference at the end of follow-up **stratified by age** using parametric g-formula with pooled logistic regression models[a].

| | Blood lead | | Urinary cadmium | |
|---|---|---|---|---|
| | Baseline age | | Baseline age | |
| Outcomes | < 50 years | >= 50 years | < 50 years | >= 50 years |
| **All-cause mortality** | | | | |
| Number of events (%) among low level before the intervention | 59/944 (6.3%) | 118/273 (43.2%) | 60/542 (11.1%) | 62/140 (44.3%) |
| Number of events (%) among high level before the intervention | 1082/7851 (13.8%) | 3685/5752 (64.1%) | 1104/8006 (13.8%) | 3541/5592 (63.3%) |
| Adjusted risk ratio (95% CI) | 2.82 (1.02 to 5.81) | 2.34 (1.41 to 3.91) | 5.77 (1.97 to 13.39) | 4.16% (1.82 to 8.60) |
| Adjusted risk difference (95% CI) | +1.38% (0.06 to 3.78) | +5.73% (3.14 to 14.15) | +2.25% (1.32 to 5.65) | +9.35% (5.66 to 24.93) |
| **Cardiovascular mortality** | | | | |
| Number of events (%) among low level before the intervention | 8/944 (0.9%) | 27/273 (9.9%) | 12/542 (2.2%) | 18/140 (12.9%) |
| Number of events (%) among high level before the intervention | 228/7851 (2.9%) | 1195/5752 (20.8%) | 233/8006 (2.9%) | 1172/5592 (21.0%) |
| Adjusted risk ratio (95% CI) | 0.78 (0.07 to 6.38) | 3.18 (1.40 to 7.46) | 2.74 (1.08 to 38.06) | 2.43 (1.21 to 5.02) |
| Adjusted risk difference (95% CI) | -0.06% (-2.62 to 0.54) | +2.11% (0.98 to 5.78) | +0.22% (0.04 to 1.22) | +1.73% (0.49 to 5.62) |
| **Cancer mortality** | | | | |
| Number of events (%) among low level before the intervention | 24/944 (2.5%) | 25/273 (9.2%) | 12/542 (2.2%) | 10/140 (7.1%) |
| Number of events (%) among high level before the intervention | 277/7851 (3.5%) | 750/5752 (13.0%) | 286/8006 (3.6%) | 721/5592 (12.9%) |
| Adjusted risk ratio (95% CI) | 5.36 (1.01 to 21.15) | 1.85 (0.65 to 6.48) | 3.13 (0.58 to 12.73) | 2.09 (0.80 to 9.39) |
| Adjusted risk difference (95% CI) | +0.80% (0.01 to 2.38) | 0.74% (-0.81 to 3.23) | +0.54% (-0.27 to 2.42) | +0.88% (-0.42 to 4.00) |

[a]200 iterations were performed for bootstrapping the estimates 95% confidence interval. Low level: 5th percentiles of blood lead (0.70 µg/dL or 0.03 umol/L) and urinary cadmium (0.04 µg/g) distributions. High level: 95th percentiles of blood lead (9.70 µg/dL or 0.47 umol/L) and urinary cadmium (1.63 µg/g) distributions.

**A** Blood lead: Women

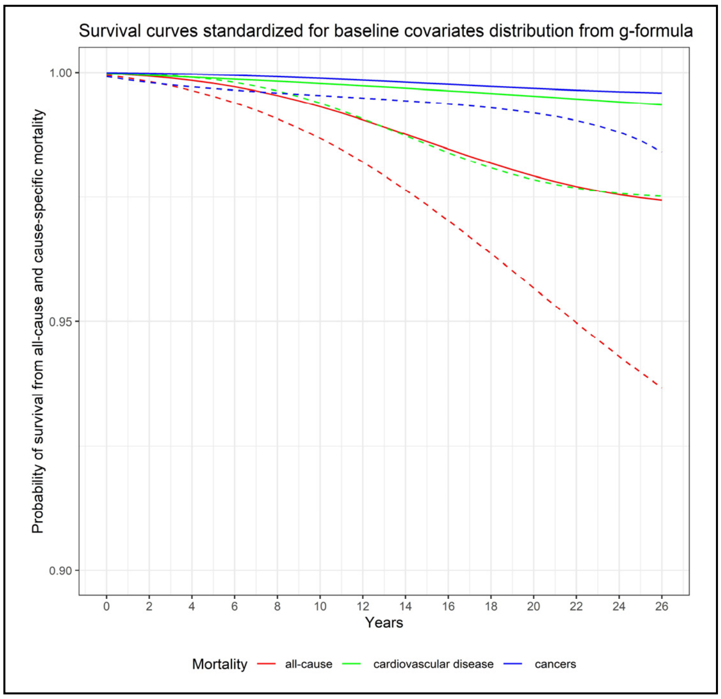


**B** Blood lead: Men

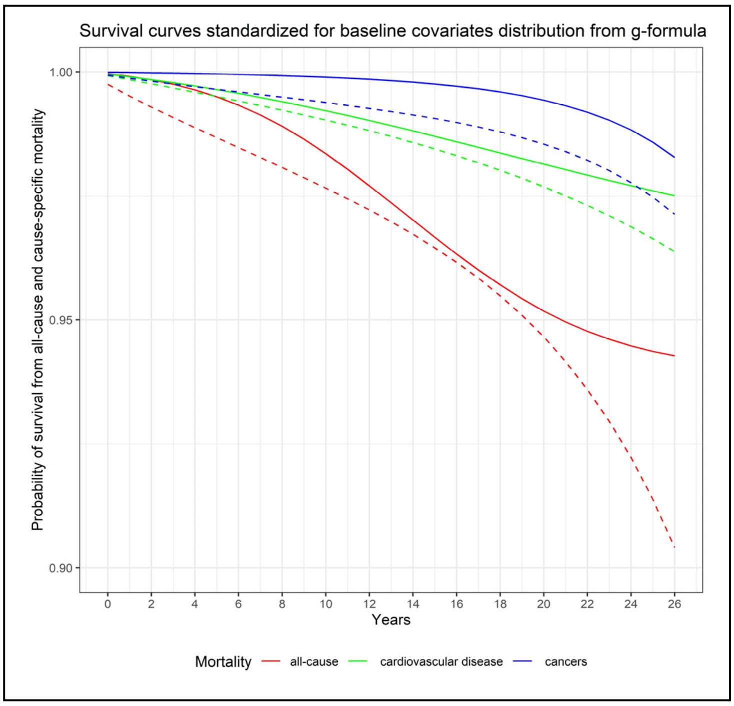


**C** Urinary cadmium: Women

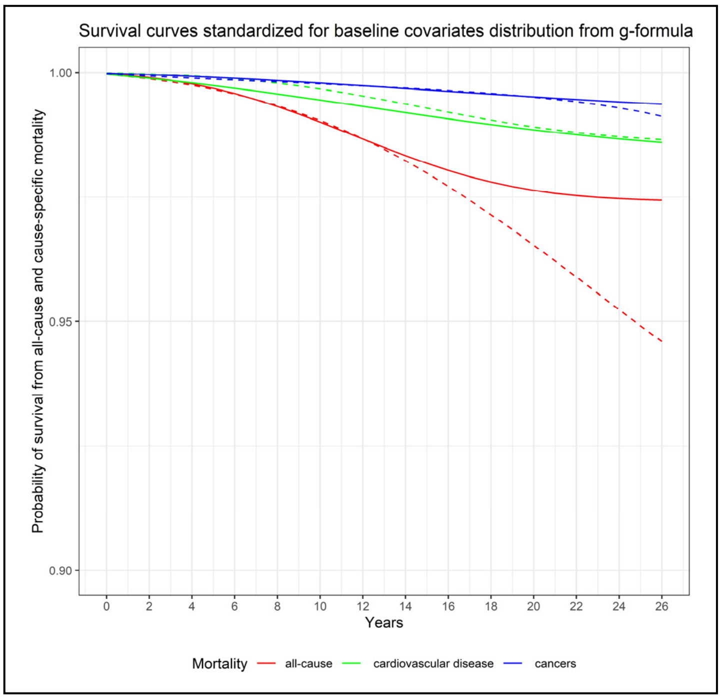


**D** Urinary cadmium: Men

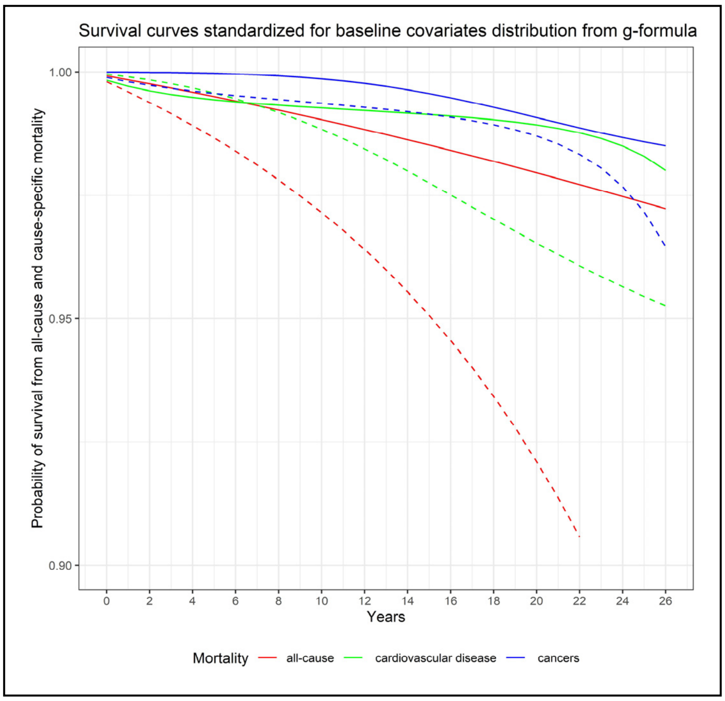


**Figure S3.** Adjusted all-cause and specific-cause of mortality risk stratified by sex using parametric g-formula with pooled logistic regression models. The solid line represents the percentile 5 (0.70 µg/dL for lead and 0.04 µg/g for cadmium) and the dashed lines the percentile 95 (9.70 µg/dL for lead and 1.63 µg/g for cadmium). Robust 95% confidence intervals (CIs) for each exposure category estimated by bootstrapping (in the pooled logistic regression model**)** are presented in Table S2.

.

Table S2: Adjusted all-cause and specific-cause of mortality risk ratio and risk difference at the end of follow-up **stratified by sex** using parametric g-formula with pooled logistic regression models[a]

| | Blood lead | | | Urinary cadmium | | |
|---|---|---|---|---|---|---|
| **Outcomes** | **Women** | **Men** | | **Women** | **Men** | |
| **All-cause mortality** | | | | | | |
| Number of events (%) among low level before the intervention | 146/1060 (13.8%) | 31/157 (19.8%) | | 52/296 (17.8%) | 70/386 (18.1%) | |
| Number of events (%) among high level before the intervention | 2332/7424 (31.4%) | 2435/6179 (39.4%) | | 2067/7073 (29.2%) | 2578/6525 (39.5%) | |
| Adjusted risk ratio (95% CI) | 2.22 (1.01 to 3.70) | 1.65 (0.99 to 2.32) | | 2.11 (0.90 to 5.02) | 5.11 (2.20 to 9.95) | |
| Adjusted risk difference (95% CI) | +3.13% (0.03 to 7.31) | +4.01% (-0.21 to 8.79) | | +2.93% (-0.65 to 10.03) | +10.06% (6.56 to 18.06) | |
| P-heterogeneity | | | 0.622 | | 0.098 | 0.247 |
| **Cardiovascular mortality** | | | | | | |
| Number of events (%) among low level before the intervention | 29/1060 (2.7%) | 6/157 (3.8%) | | 13/296 (4.4%) | 17/386 (4.4%) | |
| Number of events (%) among high level before the intervention | 690/7424 (9.3%) | 733/6179 (11.9%) | | 618/7073 (8.7%) | 787/6525 (12.1%) | |
| Adjusted risk ratio (95% CI) | 3.24 (1.19 to 6.42) | 1.37 (0.71 to 2.70) | | 0.97 (0.37 to 2.65) | 2.34 (1.02 to 6.52) | |
| Adjusted risk difference (95% CI) | +1.46% (0.28 to 3.95) | +1.01% (-1.57 to 4.19) | | -0.04% (-2.24 to 1.72) | +2.59% (0.07 to 7.13) | |
| P-heterogeneity | | | 0.202 | | 0.032 | 0.515 |
| **Cancer mortality** | | | | | | |

| | | | | | | |
|---|---|---|---|---|---|---|
| Number of events (%) among low level before the intervention | 45/1060 (4.3%) | 4/157 (2.6%) | | 15/296 (5.1%) | 7/386 (1.8%) | |
| Number of events (%) among high level before the intervention | 487/7424 (6.6%) | 540/6179 (8.7%) | | 435/7073 (6.2%) | 572/6525 (8.8%) | |
| Adjusted risk ratio (95% CI) | 3.20 (0.78 to 10.28) | 1.57 (0.56 to 5.17) | | 1.38 (0.46 to 5.67) | 2.33 (0.45 to 11.48) | |
| Adjusted risk difference (95% CI) | +0.91% (-0.23 to 3.42) | +1.09% (-2.05 to 4.60) | | +0.25% (-0.72 to 1.80) | +1.92% (-0.03 to 6.76) | |
| P-heterogeneity | | | 0.206 | | 0.102 | 0.628 |

[a]200 iterations were performed for bootstrapping the estimates 95% confidence interval

Low level: 5th percentiles of blood lead (0.70 µg/dL or 0.03 umol/L) and urinary cadmium (0.04 µg/g) distributions

High level: 95th percentiles of blood lead (9.70 µg/dL or 0.47 umol/L) and urinary cadmium (1.63 µg/g) distributions.

Table S3: **Unweighted** adjusted all-cause and specific-cause of mortality risk ratio and risk difference of metals percentile 95 and 5 values using parametric g-formula with weighted pooled logistic regression models[a]

| Outcomes | Blood lead | Urinary cadmium |
|---|---|---|
| **All-cause mortality** | | |
| Number of events (%) among low level before the intervention | 177/1217 (14.5%) | 122/682 (17.9%) |
| Number of events (%) among high level before the intervention | 4767/13603 (35.0%) | 4645/13598 (34.2%) |
| Adjusted risk ratio (95% CI) | 1.60 (1.20 to 2.40) | 2.70 (2.07 to 4.87) |
| Adjusted risk difference (95% CI) | +3.91% (1.84 to 8.36) | +6.90% (6.00 to 11.88) |
| **Cardiovascular mortality** | | |
| Number of events (%) among low level before the intervention | 35/1217 (2.9%) | 30/682 (4.4%) |
| Number of events (%) among high level before the intervention | 1423/13603 (10.5%) | 1405/13598 (10.3%) |
| Adjusted risk ratio (95% CI) | 1.02 (0.52 to 2.80) | 2.64 (1.47 to 5.58) |
| Adjusted risk difference (95% CI) | +0.69% (-2.72 to 3.85) | +0.22% (1.13 to 5.32) |
| **Cancer mortality** | | |
| Number of events (%) among low level before the intervention | 49/1217 (4.0%) | 22/682 (3.2%) |
| Number of events (%) among high level before the intervention | 1027/13603 (7.6%) | 1007/13598 (7.4%) |
| Adjusted risk ratio (95% CI) | 2.41 (0.70 to 3.69) | 3.30 (1.03 to 7.00) |
| Adjusted risk difference (95% CI) | +0.49% (-1.66 to 2.42) | +1.11% (0.05 to 3.23) |

[a]200 iterations were performed for bootstrapping the estimates 95% confidence interval

low level: 5$^{th}$ percentiles of blood lead (0.70 μg/dL or 0.03 umol/L) and urinary cadmium (0.04 μg/g) distributions

high level: 95$^{th}$ percentiles of blood lead (9.70 μg/dL or 0.47 umol/L) and urinary cadmium (1.63 μg/g) distributions

**Table S4.** Adjusted all-cause and specific-cause of mortality risk ratio and risk difference of metals percentile 95 and 5 values using parametric g-formula with pooled logistic regression models[a] , **model adjusted for tobacco smoke measured by cotinine level and pack-years of cumulative active smoking.**

| Outcomes | Blood lead | Urinary cadmium |
|---|---|---|
| **All-cause mortality** | | |
| Adjusted risk ratio (95% CI) | 2.36 (0.96 to 4.92) | 3.72 (1.82 to 6.48) |
| Adjusted risk difference (95% CI) | +4.00% (-0.28 to 10.86) | +5.94% (3.92 to 11.19) |
| **Cardiovascular mortality** | | |
| Adjusted risk ratio (95% CI) | 2.95 (1.06 to 6.77) | 6.12 (1.85 to 21.87) |
| Adjusted risk difference (95% CI) | +1.22% (0.30 to 3.51) | +2.37% (1.33 to 6.07) |
| **Cancer mortality** | | |
| Adjusted risk ratio (95% CI) | 1.51 (0.18 to 7.68) | 5.08 (1.04 to 29.39) |
| Adjusted risk difference (95% CI) | +0.53% (-0.03 to 4.48) | +1.46% (0.07 to 4.17) |

[a]200 iterations were performed for bootstrapping the estimates 95% confidence interval

low level: 5$^{th}$ percentiles of blood lead (0.70 μg/dL or 0.03 umol/L) and urinary cadmium (0.04 μg/g) distributions

high level: 95$^{th}$ percentiles of blood lead (9.70 μg/dL or 0.47 umol/L) and urinary cadmium (1.63 μg/g) distributions

**Table S5.** Mid-follow-up (13 years) adjusted all-cause and specific-cause of mortality risk ratio and risk difference of metals percentile 95 and 5 values using parametric g-formula with pooled logistic regression models[a].

| Outcomes | Blood lead | Urinary cadmium |
|---|---|---|
| **All-cause mortality** | | |
| Adjusted risk ratio (95% CI) | 3.17 (1.26 to 3.24) | 1.80 (1.76 to 4.95) |
| Adjusted risk difference (95% CI) | +0.85% (0.44 to 4.28) | +2.03% (1.14 to 5.47) |
| **Cardiovascular mortality** | | |
| Adjusted risk ratio (95% CI) | 2.10 (1.09 to 3.65) | 0.86 (0.74 to 2.98) |
| Adjusted risk difference (95% CI) | +0.47% (0.01 to 1.96) | +0.27% (-0.45 to 1.69) |
| **Cancer mortality** | | |
| Adjusted risk ratio (95% CI) | 5.27 (1.18 to 8.38) | 3.51 (0.67 to 4.34) |
| Adjusted risk difference (95% CI) | +0.35% (0.10 to 1.94) | +0.18% (-0.41 to 1.39) |

[a]200 iterations were performed for bootstrapping the estimates 95% confidence interval

low level: 5th percentiles of blood lead (0.70 µg/dL or 0.03 umol/L) and urinary cadmium (0.04 µg/g) distributions

high level: 95th percentiles of blood lead (9.70 µg/dL or 0.47 umol/L) and urinary cadmium (1.63 µg/g) distributions.

toxics MDPI

**Table S6.** Mid-follow-up (13 years) adjusted all-cause and specific-cause of mortality risk ratio and risk difference of metals extreme quartiles using parametric g-formula with pooled logistic regression models[a].

| Outcomes | Blood lead | Urinary cadmium |
|---|---|---|
| **All-cause mortality** | | |
| Adjusted risk ratio (95% CI) | 1.76 (1.16 to 3.69) | 1.83 (1.75 to 5.65) |
| Adjusted risk difference (95% CI) | +0.42% (0.38 to 7.45) | +2.42% (1.03 to 11.22) |
| **Cardiovascular mortality** | | |
| Adjusted risk ratio (95% CI) | 3.82 (1.42 to 24.26) | 7.33 (3.06 to 100.04) |
| Adjusted risk difference (95% CI) | +0.91% (0.46 to 19.84) | +3.74% (2.85 to 50.26) |
| **Cancer mortality** | | |
| Adjusted risk ratio (95% CI) | 2.86 (1.07 to 15.31) | 3.23 (0.47 to 34.71) |
| Adjusted risk difference (95% CI) | +0.21% (0.08 to 9.42) | +0.12% (-0.55 to 18.23) |
